# A Case of Dengue Fever With Multiserotype IgG Positivity

**DOI:** 10.7759/cureus.72234

**Published:** 2024-10-23

**Authors:** Noah R Wheaton, Davon T Lee, Samrawit W Zinabu, Courtui Sims, Syneja Richards, Jessica Ray, Betelehem Atalay, Ahmad Mohammed, Miriam B Michael

**Affiliations:** 1 Internal Medicine, Howard University Hospital, Washington, DC, USA; 2 Internal Medicine, University of Maryland, Baltimore, USA

**Keywords:** aedes mosquito, dengue fever, flavivirus, global warming and rising temperature, travelers

## Abstract

Dengue fever is a rapidly spreading mosquito-borne viral disease, increasingly endemic in tropical and subtropical regions. This case report details the clinical presentation of a 52-year-old male who developed severe symptoms shortly after returning from a two-week trip to El Salvador, an area known for dengue endemicity. The patient presented with a five-day history of high-grade fever, malaise, diarrhea, dizziness, and intermittent blurry vision, accompanied by chills, dry cough, headaches, and generalized pain. Physical examination revealed petechiae on the lower extremities, and the Rumpel-Leede test was positive, indicating capillary fragility and suggesting thrombocytopenia consistent with dengue infection. Supportive care, including antipyretics, hydration, and electrolyte management, resolved symptoms by day four, allowing the patient to be discharged. This case underscores the importance of considering dengue in patients with relevant travel history and highlights the increasing global reach of this vector-borne disease.

## Introduction

Over the past three decades, dengue has rapidly spread and emerged as the most prevalent mosquito-borne viral disease in humans, with cases continuing to rise globally. Although commonly associated with tropical and subtropical regions, dengue can also be transmitted in temperate climates. Currently, dengue is endemic in Mexico, most Latin American countries, and parts of the Caribbean, potentially reestablishing itself as an endemic disease in the United States [1]. This looming threat requires immediate attention and action.

Dengue is a mosquito-borne virus and the leading cause of arthropod-borne viral diseases worldwide. It is primarily transmitted by several species of *Aedes* mosquitoes, including *Aedes aegypti*, *Aedes polynesiensis*, *Aedes scutellaris*, and *Aedes albopictus* [2]. Dengue fever is caused by one of the four distinct serotypes (DENV-1 to DENV-4) of the dengue virus, which is a single-stranded RNA (ssRNA) virus with a positive-sense (5'-to-3') RNA. The dengue virus, which belongs to the Flaviviridae family, shares similarities with other viruses such as yellow fever, West Nile, Japanese encephalitis, and tick-borne encephalitis [3]. The antigenic diversity of DENV strains is linked to variations in disease severity and the intensity of dengue outbreaks [4]. The incidence of dengue fever has dramatically increased over the past few decades, possibly due to increased global travel, leading to the infection becoming endemic in some regions of the world.

A complex interaction between host and viral factors shapes the presentation of dengue fever. Common symptoms include fever, rash, nausea, vomiting, and body aches. However, a severe complication of dengue, known as dengue shock syndrome (DSS), is marked by significant bleeding from the blood vessels and shock, with mortality rates reaching up to 20% if left untreated [5]. Severe dengue fever is commonly associated with secondary infections caused by different dengue virus serotypes in endemic areas, although severe cases can also occur with a single serotype infection. The diagnosis of dengue is confirmed through methods such as serological testing, viral culture, or polymerase chain reaction [2].

## Case presentation

A 52-year-old male presented to the emergency department with a five-day history of malaise, three days of diarrhea, dizziness, two episodes of syncope, a persistent fever ranging from 102 to 104°F, and intermittent blurry vision, after returning from a two-week trip to El Salvador. He noted that his fever temporarily subsided for approximately two hours after acetaminophen administration, with temperatures spiking at night. Additional symptoms included chills, dry cough, headaches, and generalized body pain. His medical history includes hypertension, prediabetes, and fatty liver disease. The patient had not visited his home country in 20 years, and during his stay in El Salvador, he visited many houses and the homes of his family and neighbors. Given his recent travel to an endemic region and the severity of his symptoms, mosquito-borne diseases such as dengue, chikungunya, and malaria were considered differential diagnoses.

**Figure 1:**
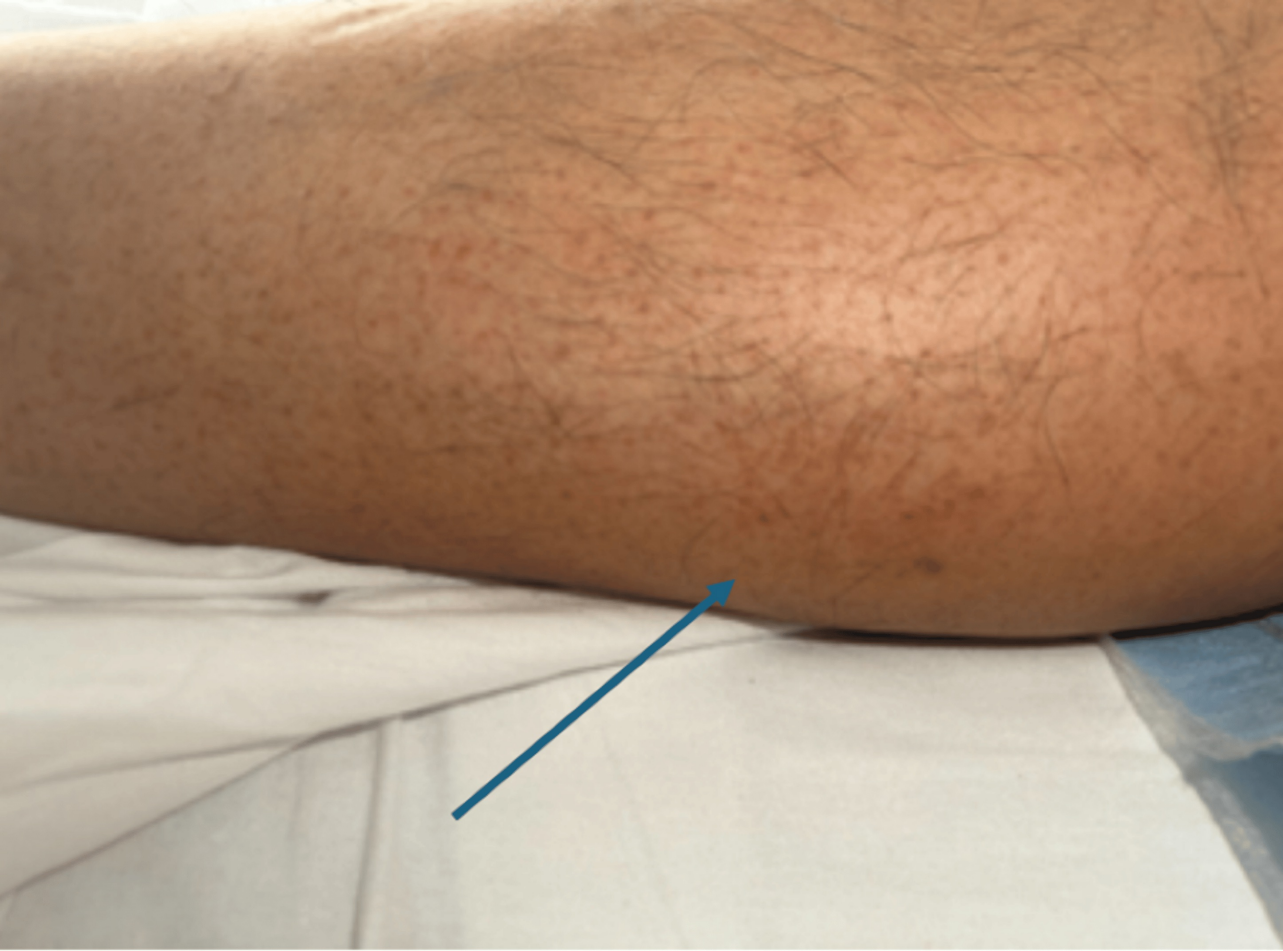
Petichial rash on the bilateral lower extremities

**Figure 2:**
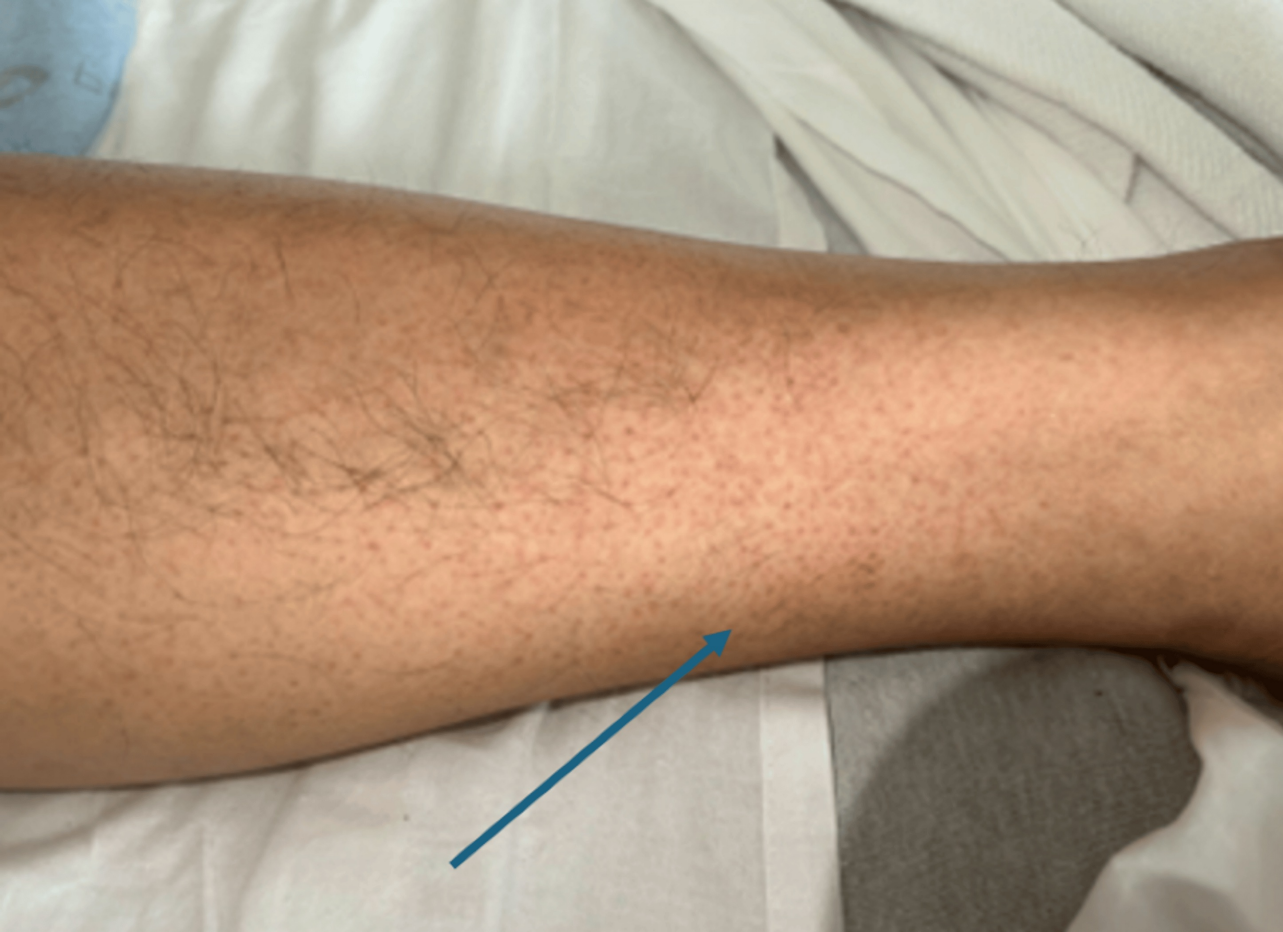
Petichial rash on the bilateral lower extremities

During the initial assessment, the patient was alert and oriented. His vital signs revealed a blood pressure of 110/66 mmHg, a temperature of 106°F, a respiratory rate of 18 breaths per minute, a pulse of 102 beats per minute, and an oxygen saturation of 98%. A petechial rash was observed on both the lower extremities (Figure 1 and Figure 2). The Rumpel-Leede test, an indicator of capillary fragility often used in dengue diagnosis, was positive. Laboratory investigations revealed elevated liver enzymes with aspartate aminotransferase at 85 IU/L and alanine aminotransferase at 64 IU/L, thrombocytopenia with a platelet count of 56,000/µL, hemoglobin of 15.6 g/dL, and a hematocrit of 45.8% (Table 1). Dengue serology showed positive IgG antibodies for all four dengue virus serotypes. The patient was treated with supportive care, including oral acetaminophen, Ringer’s lactate solution, and correction of electrolyte imbalances. He was discharged on the fourth day of hospitalization.

**Table 1:** Lab tests with corresponding values upon arrival, during admission, and at discharge alongside time of lowest value INR: international normalized ratio.

Lab test	Normal range	Value upon arrival	Lowest value during admission	Value at discharge	Time of lowest value
Sodium (mEq/L)	135-145	133	133	140	On arrival
Creatinine (mg/dL)	0.7-1.3	1.5	1.5	0.82	On arrival
Calcium (mg/dL)	8.8-10.2	8.2	7.6	8.8	Three hours post-arrival
Alanine transaminase (IU/L)	10-40	64	54	54	3 hours post-arrival
Aspartate transaminase (IU/L)	10-40	85	75	75	3 hours post-arrival
Platelet concentration (x10E9)	150-450	56	28	253	30 hours post-arrival
Hemoglobin (g/dL)	12-18	15.6	13.2	13.6	48 hours post-arrival
Hematocrit (%)	36-50	45.8	37.9	41.3	48 hours post-arrival
Lipase (IU/L)	0-140	137	-	-	-
Activated partial thromboplastin time (seconds)	21-35	41.4	-	-	-
INR (seconds)	0.8-1.2	0.98	-	-	-

## Discussion

The clinical presentation of dengue can vary widely, ranging from asymptomatic cases to severe manifestations. Common symptoms include a self-limiting fever, headache, myalgia, and rash, typically lasting 5-7 days, often accompanied by leukopenia and thrombocytopenia. In more severe cases, patients may present with nausea, vomiting, petechiae, and, in some instances, life-threatening conditions such as DHF or DSS [3,6].

In this case, the patient’s symptoms began shortly after returning from El Salvador and progressively worsened, including malaise, diarrhea, dizziness, syncope, continuous high-grade fever, and intermittent blurry vision. The development of petechiae and a positive Rumpel-Leede test suggest dengue's characteristic capillary fragility and thrombocytopenia. The onset and nature of these symptoms raised significant concerns for travel-related infectious diseases, particularly given the patient’s recent travel to a region where dengue fever is endemic. El Salvador, located in Central America, is known for its dengue prevalence. Thrombocytopenia, hypocalcemia, elevated liver enzymes, and elevated creatinine are common findings in dengue cases and are attributed to several pathophysiological mechanisms, including increased vascular permeability, dehydration, and direct tissue damage caused by the virus. These clinical findings, positive serological evidence for IgG antibodies to all four dengue virus serotypes, and travel history confirmed the diagnosis. Although dengue IgG positivity indicates prior exposure or infection, it may also suggest reinfection, heightening the risk of severe dengue or DHF. Given that the patient had not traveled to El Salvador in 20 years, the positive IgG for all four dengue serotypes may suggest that the current infection could involve multiserotype exposure to the four dengue virus serotypes. This case underscores the importance of considering epidemiological factors, especially travel history, in the differential diagnosis.

As previously noted, dengue is widespread not only in Mexico but also across many Latin American countries and parts of the Caribbean. The rise in global temperatures has contributed to the changing patterns of dengue endemicity. Dengue is transmitted to humans through the bite of an infected *Aedes* mosquito. As temperatures increase in the Northern Hemisphere, areas that historically did not support the survival of adult *Aedes* mosquitoes are now becoming suitable habitats, leading to a rise in vector-borne diseases, including dengue [6,7]. Given the increasing incidence of dengue, it is imperative to focus on preventative strategies rather than solely reactive measures to address this growing public health concern. The Centers for Disease Control and Prevention (CDC) has provided recommendations for preventative strategies against dengue virus transmission. Such recommendations include utilizing insect repellent and wearing loose-fitting, long-sleeved shirts and pants to prevent mosquito bites [8]. In addition, individuals who travel to endemic areas should review country-specific travel recommendations and health notices [9].

## Conclusions

Although this patient was successfully managed and discharged without further complications, the increasing incidence of dengue globally, even in nontropical regions, calls for robust public health measures, including vector control strategies, public education, and early detection systems. Clinicians must remain vigilant for dengue, particularly in patients with recent travel history, as early diagnosis and treatment can mitigate the risk of severe complications like DHF or DSS. 
